# The Targeting of Plasmalemmal Ceramide to Mitochondria during Apoptosis

**DOI:** 10.1371/journal.pone.0023706

**Published:** 2011-08-19

**Authors:** Eduard B. Babiychuk, Alexander P. Atanassoff, Katia Monastyrskaya, Christina Brandenberger, Daniel Studer, Catherine Allemann, Annette Draeger

**Affiliations:** Institute of Anatomy, University of Bern, Bern, Switzerland; Boston University, United States of America

## Abstract

Ceramide is a key lipid mediator of cellular processes such as differentiation, proliferation, growth arrest and apoptosis. During apoptosis, ceramide is produced within the plasma membrane. Although recent data suggest that the generation of intracellular ceramide increases mitochondrial permeability, the source of mitochondrial ceramide remains unknown. Here, we determine whether a stress-mediated plasmalemmal pool of ceramide might become available to the mitochondria of apoptotic cells. We have previously established annexin A1—a member of a family of Ca^2+^ and membrane-binding proteins—to be a marker of ceramide platforms. Using fluorescently tagged annexin A1, we show that, upon its generation within the plasma membrane, ceramide self-associates into platforms that subsequently invaginate and fuse with mitochondria. An accumulation of ceramide within the mitochondria of apoptotic cells was also confirmed using a ceramide-specific antibody. Electron microscopic tomography confirmed that upon the formation of ceramide platforms, the invaginated regions of the plasma membrane extend deep into the cytoplasm forming direct physical contacts with mitochondrial outer membranes. Ceramide might thus be directly transferred from the plasma membrane to the mitochondrial outer membrane. It is conceivable that this “kiss-of-death” increases the permeability of the mitochondrial outer membrane thereby triggering apoptosis.

## Introduction

An increased permeability of the mitochondrial outer membrane is implicated in the early stages of apoptosis [1]. The release of pro-apototic proteins from mitochondria can be mediated by the activation of Bax [2]. Recent data suggest that Bax preferentially inserts into mitochondria via ceramide-enriched microdomains, however, details of its targeting mechanism have not been resolved [3]–[5]. On the other hand, an increase in mitochondrial permeability has been ascribed to ceramide itself: Accumulation of mitochondrial ceramide might promote the formation of membrane channels and the subsequent release of proapoptotic proteins from the mitochondrial intermembrane space [6].

Mitochondria do not participate in interorganellar vesicular trafficking. Hence, during the onset of apoptosis, ceramide has to be either produced on-site or rapidly transported to the mitochondria from other sources. Sphingomyelinase-dependent hydrolysis of sphingomyelin and *de novo* synthesis are the two major pathways of ceramide biosynthesis [7]–[9]. The *de novo* production of ceramide is confined to the endoplasmic reticulum, and the mechanism whereby the newly synthesized ceramide is transferred to the mitochondria is unknown. A recent study has provided evidence for the existence of a mitochondrial-associated neutral sphingomyelinase [10]. It is thus possible that mitochondrial sphingolipids [11], [12] are metabolically converted to ceramide *in situ* and are locally assembled into ceramide channels within the mitochondrial outer membrane [13], [14]. However, it is particularly the plasmalemmal ceramide, which is generated by the activity of acid sphingomyelinase that is implicated in the induction of apoptosis [14]. Moreover, a soluble ceramide transfer protein (CERT) has been described [15], which could transport ceramide to the mitochodria from the Golgi apparatus. Thus, although an increase in mitochondrial ceramide in response to the initiation of apoptosis has been documented [4], [16], the source of mitochondrial ceramide and its mechanism of entry are unknown.

Massive, calcium-activated endocytosis of the plasma membrane without involvement of classical endocytic proteins is a recently described phenomenon and is closely associated with the formation of ceramide during cellular stress [17]–[20]. We have investigated interorganellar membrane contacts during this process.

Using confocal microscopy in living cells and electron microscopic tomography of unfixed, high-pressure frozen cells, we have demonstrated that ceramide platforms, initially formed within the plasma membrane of apoptotic cells, are internalized and come into close contact with the mitochondrial outer membrane. It is conceivable that ceramide is exchanged at these contact points, and that this triggers the release of pro-apoptotic molecules.

## Materials and Methods

### Reagents and antibodies

The mouse polyclonal antibody against ceramide [21] was purchased from Glycobiotech GmbH (Kuekels, Germany). The Living Colours Fluorescent protein vectors peCFP-N1, peYFP-N1 and pDsRed-Mito were obtained from Clontech Europe (St Germain-en-Laye, France), and the restriction endonucleases, Taq polymerase and T4 DNA ligase from New England Biolabs (Bioconcept, Allschwil, Switzerland). Other reagents were purchased from Sigma (Buchs, Switzerland).

### Cell culture and transfections

The coding sequences of annexins A1 and A6 were cloned into the Living Colours Fluorescent protein vectors yellow-fluorescent protein (YFP), green fluorescent protein (GFP) and cyan fluorescent protein (CFP), following the PCR amplification from human bladder smooth muscle cDNA [22].

Jurkat T cells and a human monocyte cell line (THP-1) were cultured in RPMI medium containing 5% calf serum and penicillin/streptomycin. The cells were grown in 5% CO_2_ at 37°C in a humidified incubator. They were transiently transfected with plasmids by electroporation (BioRad) and analysed after incubation at 37°C for 48 hours.

### Confocal imaging

Annexin A1-YFP, annexin A1-GFP, annexin A6-CFP and dsRed-Mito were transiently expressed in Jurkat T-cells and THP-1 cells. The cells, which were allowed to settle on glass coverslips, were mounted in a perfusion chamber in Tyrode's solution (140 mM NaCl, 5 mM KCl, 1 mM MgCl_2_, 10 mM glucose, 10 mM HEPES, pH = 7.4) to which was added 2 mM CaCl_2_. At time point zero, the cells were challenged either with Streptolysin O [SLO (100 ng/ml)] or with ionomycin (5 µM). The fluorescence signal was recorded using a ×100 oil immersion lens in an Axiovert 200 M microscope with a laser scanning module LSM 510 META (Zeiss, Germany).

### Immunofluorescence analysis

Immunofluorescence was performed as previously described [22], [23], with the following modifications for non-adherent cells: Following centrifugation at 100 g, the pelleted Jurkat T and the THP-1 cells were suspendend in Tyrode's solution. Some cells were treated with a cell permeant, mitochondrium-specific dye [Mito-ID™ Red (Enzo Life Sciences, Lausen, Switzerland)] according to the manufactorer's instructions, followed by SLO (100 ng/ml) or ionomycin (5 µM) treatment in suspension in Tyrode's buffer containing 2 mM Ca^2+^ for 15 min. The cells were allowed to settle on coverslips, chemically fixed with 4% paraformaldehyde in Tyrode's solution for 5 min and finally permeabilized with 0.5% Triton X-100 (in Tyrode's solution) for 30 s at ambient temperature.

The colocalization of mitochondria with ceramide reporter protein annexin A1-GFP after stimulation of cells with ionomycin was quantified in Jurkat cells in 4 independent experiments and with SLO in 2 independent experiments. Identical stimulations were conducted in THP-1 cells with ionomycin (5 independent experiments) and with SLO (2 independent experiments). The percentage of colocalization of annexin A1-GFP with Mito-ID™ Red in each cell was established by calculating the ratio of merged pixels (red/green) to the total pixels, showing a fluorescent intensity in either of the channels above the background, within the observation field containing at least 30 cells.

### Ultrastructural investigations

G_m1_ ganglioside receptors on the plasmalemmal surface of Jurkat T-cells and THP-1 cells were labelled with horseradish-peroxidase-tagged cholera toxin B (HRP-CTX B), and then stimulated either with SLO (100 ng/ml) or with ionomycin (5 µM) for 20 min. The peroxidase was developed with 0.02% diaminobenzidine in Tyrode's buffer containing 2 mM Ca^2+^ and 0.01% H_2_O_2_ for 15 min. The samples were washed in Tyrode's solution containing 2 mM CaCl_2_ and the samples were immediately subjected to a pressure of 210 MPa [using a Leica EMPACT instrument (FEI Company, The Netherlands)] and simultaneously cooled down to −196°C by a double jet of liquid nitrogen [24]. Freeze substitution was performed as described previously [25]. In brief, the samples were dehydrated at −90°C, treated with 2% osmium tetroxide at −70°C and embedded in Epon at temperatures between −50°C and ambient temperature. 250 nm-thick-sections were subjected to electron microscopic tomography in a Tecnai F20 transmission electron microscope, which was equipped with a GIF Tridiem energy filter and an Ultrascan 1000 CCD camera (Gatan, Pleasanton, USA). The samples were secured in a dual tilt Fischione specimen holder (Fischione Instruments, USA) and tomograms were recorded continuously over a tilt-angle shift ranging −70° to +70°. To correct for the missing wedge (−90°to −70°/+90°to +70°) dual tilt axis acquisition was performed with an angle difference of 90°. Image processing and 3D-stack reconstruction were achieved using the Inspect 3D software V.3.0. (FEI Company).

Plasmalemmal invaginations were counted in ultrathin sections of Jurkat cells (n = 1800) in 6 independent experiments (∼25 randomly selected visual fields/experiment). Plasmalemmal-mitochondrial proximity was considered evident at ≤0.2 µm distance between the 2 organelles.

## Results

### Intracellular Ca^2+^-overload induces an accumulation of mitochondrial ceramide

Ca^2+^-overload is a potent pro-apoptotic stimulus [26], [27], which leads to the hydrolysis of sphingomyelin and the formation of ceramide at the plasma membrane [18].

The observation that ceramide was present within mitochondria during the early stages of apoptosis [28] prompted us to investigate a potential transport of ceramide from the plasmalemma to mitochondria.

**Figure 1:**
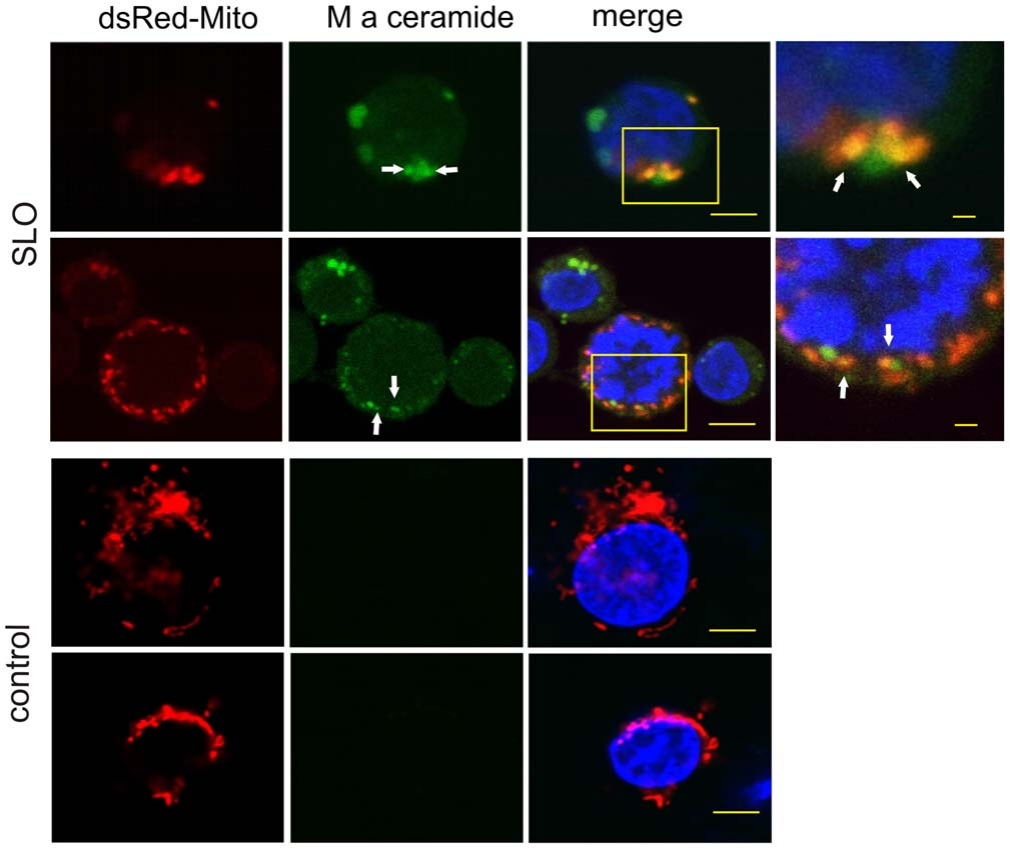
Ca^2+^-overload leads to the appearance of ceramide in mitochondria. The mitochondrial marker dsRed-Mito (red) was transiently expressed in Jurkat T-cells, which were either stimulated with SLO or left untreated (control). Subsequently, the cells were chemically fixed and treated with a polyclonal antibody against ceramide (M a ceramide; green) and Hoechst stain as a nuclear marker (blue). Confocal micrographs revealed ceramide platforms and their partial colocalization with mitochondria in stimulated cells (yellow, arrows). The boxed areas are enlarged. No ceramide platforms are present in unstimulated cells. Bars in the merged images = 3 µm, enlarged boxed areas = 1 µm.

An intracellular Ca^2+^-overload was induced in Jurkat T-cells or in THP-1 cells by treating them with the pore-forming toxin SLO or with ionomycin [18]. Immunofluorescence microscopy of cells permeabilized with SLO revealed a partial colocalization of the mitochondrial marker dsRed-Mito with a monospecific antibody against ceramide (Fig. 1). No ceramide was detected within unstimulated (control) cells (Fig. 1).

**Figure 2:**
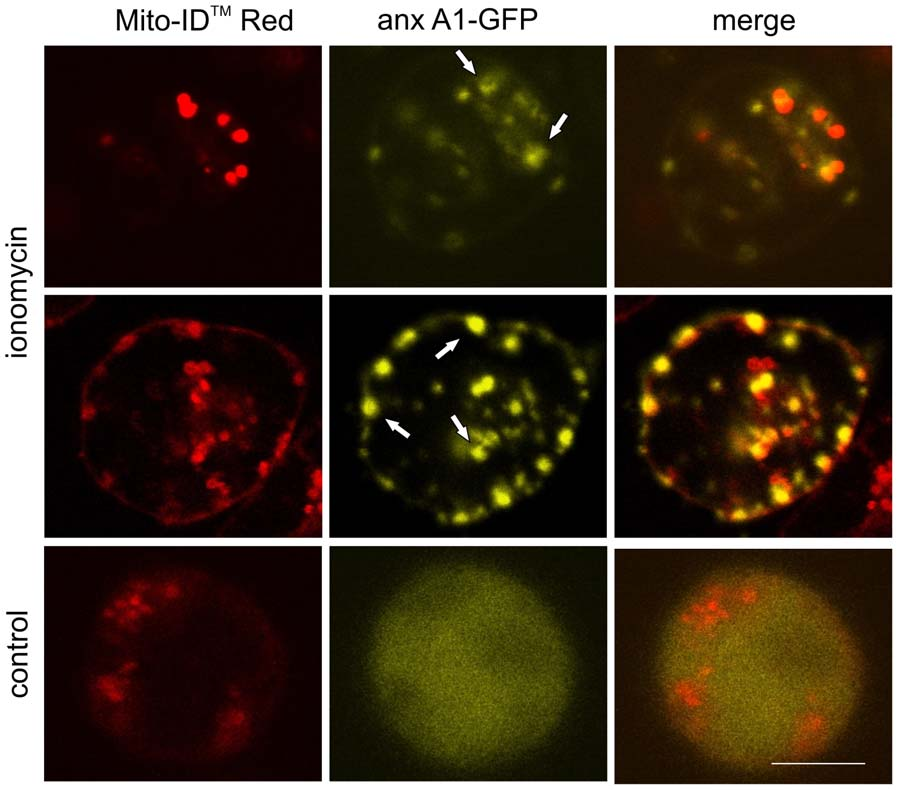
Annexin A1 partially colocalises with mitochondria after intracellular Ca^2+^-overload. Confocal micrographs of living Jurkat T-cells that had been transiently transfected with annexin A1-GFP, treated with the mitochondrial marker Mito-ID™Red (red) and stimulated with ionomycin. A partial co-localization of annexin A1 with mitochondria can be observed (arrows). The untreated cell (control) shows a homogeneous distribution of annexin A1 throughout the cytoplasm. Bar = 3 µm.

Similarly, a partial colocalization between mitochondria (labelled with Mito-ID™Red) and ceramide, labelled with the ceramide-reporter protein annexin A1 [18], [29]), was observed (Fig. 2, arrows). Within unstimulated (control) cells, annexin A1-GFP was diffusely distributed throughout the cytoplasm (Fig. 2).

Colocalization analysis revealed 29±10.8% overlap of Mito-ID™Red and annexin A1-GFP in Jurkat cells (ionomycin: n = 24; 4 independent experiments, SLO: n = 13; 2 independent experiments) and 31±17.8% STD overlap in THP-1 cells (ionomycin: n = 19; 5 independent experiments, SLO: n = 9; 2 independent experiments).

### Internalisation of plasmalemmal ceramide platforms

Upon persistent intracellular Ca^2+^-elevation, annexin A1 associates with ceramide platforms, while annexin A6 delineates the plasma membrane due to their differential lipid sensitivity of membrane binding [18], [22], [29].

**Figure 3:**
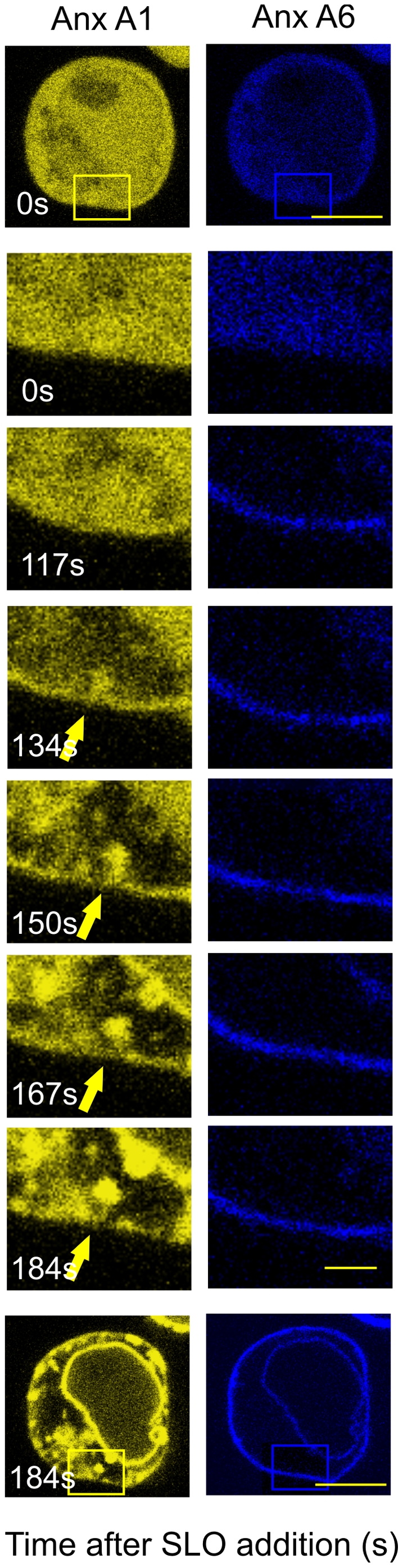
Annexin A1 invagination in a Jurkat T-cell after intracellular Ca^2+^-overload. Time-lapse sequence of confocal micrographs for a Jurkat T-cell that had been transiently transfected with annexin A1-YFP (Anx A1, yellow) and annexin A6 (Anx A6, blue) prior to stimulation with SLO. Both annexins translocate to the plasma membrane between time points 117–134. Thereafter, annexin A1 segregates from annexin A6 and coalesces into membrane platforms, which are internalised. The development of annexin A1-decorated, finger-like invaginations after Ca^2+^overload (arrows) was monitored over 3 min (time in s). Images at selected time-points are illustrated. Those at time-points zero and 184s (bars = 3 µm) are represented at lower magnification (boxed areas; bar = 1 µm) to aid orientation.

Real-time confocal microscopy of Jurkat T-cells, that had been doubly transfected with annexin A1-YFP and annexin A6-CFP shows that an increase in [Ca^2+^]_i_ led to the translocation of both annexins from the cytoplasm to the plasma membrane (Fig. 3). Initially, homogenously co-distributed, the two annexins began to segregate as the formation and self-association of ceramide progressed (Fig. 3: 134s). The accumulation of ceramide/annexin A1 platforms was followed by extensive invagination of the plasmalemma (Fig. 3: 134–150s) and their translocation from the plasma membrane to the interior of the cell (Fig. 3 & Movie S1). Annexin A6 did not participate in platform formation and was not internalised (Fig. 3 & Movie S1).

### Physical interaction between the invaginated plasma membrane and the mitochondria visualized by electron microscopic tomography

**Figure 4:**
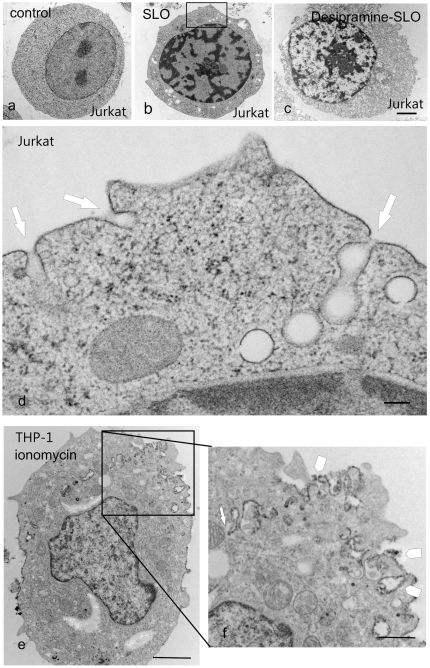
Tube-like invagination of the plasma membrane after intracellular Ca^2+^-overload. Electron micrographs of unfixed, high pressure frozen, freeze-substituted Jurkat T cells and THP-1 cells at rest (a), after stimulation with SLO (b,d) and after treatment with sphingomyelinase inhibitor desipramine before stimulation with SLO (c). (e,f ) THP-1 cells were pre-labelled with horseradish peroxidase-cholera toxin B (arrowheads) before treatment with ionomycin. a: The untreated (control) cell displays a homogeneous cytoplasm and a uniformally smooth plasmalemmal surface. b,d: The SLO-stimulated cell contains numerous cytoplasmic vacuolations, which at higher resolution [d (boxed area in b] turn out to be the profiles of tubular invaginations of the plasma membrane (arrows). c: For the inhibition of sphingomyelinase, the cells were preincubated at 37°C for 90 minutes in the presence of 50 µM desipramine prior to stimulation with SLO. e: please note deep surface-labelled plasma membrane invaginations, boxed area enlarged in (f, arrow) Bars: a,b,c,e = 1 µm, d = 0.2 µm, f = 0.5 µm.

In search of a potential physical interaction between the invaginated plasma membrane and the mitochondria, the process of invagination was monitored by electron microscopy. Since lipids are not immobilised by chemical fixation, preparation artefacts can distort lipid-driven changes in membrane curvature. In order to preserve the ultrastructure of the membranes in a near-native state, the molecules were immobilised by high-pressure freezing (210 MPa) with cooling rates which avert the formation and growth of intracellular ice crystals [24]. This process was followed by freeze substitution, which, in contrast to chemical fixation in a liquid phase, preserves the 3D framework of the constituent molecules in the absence of osmotic effects (Fig. 4). These methods combined high-speed immobilisation and preservation of cellular structures without chemical cross-linking. Thus, the ultrastructural pattern of “native” membrane internalisation could be investigated. Consistent with the lower-resolution confocal images (Fig. 3), resting Jurkat T-cells (Fig. 4a) or THP-1 cells (not shown) displayed a uniformal, smooth plasmalemmal surface. In cells, that had been exposed to a Ca^2+^-overload, tube-like invaginations were observed, branching out from numerous plasmalemmal sites and extending deep into the intracellular space (Fig. 4b boxed area enlarged in d).

An inhibitor of acid sphingomyelinase (desipramine;[30]), was used to reduce the levels of ceramide after [Ca^2+^]_i_ overload [18]. Cells treated with 50 µM desipramine for 90 min prior to exposure to SLO showed a clumpy cytoplasm and engorged intracellular organelles, but did not display plasmalemmal infoldings or disruptions (Fig. 4c, see also [18]).

**Figure 5:**
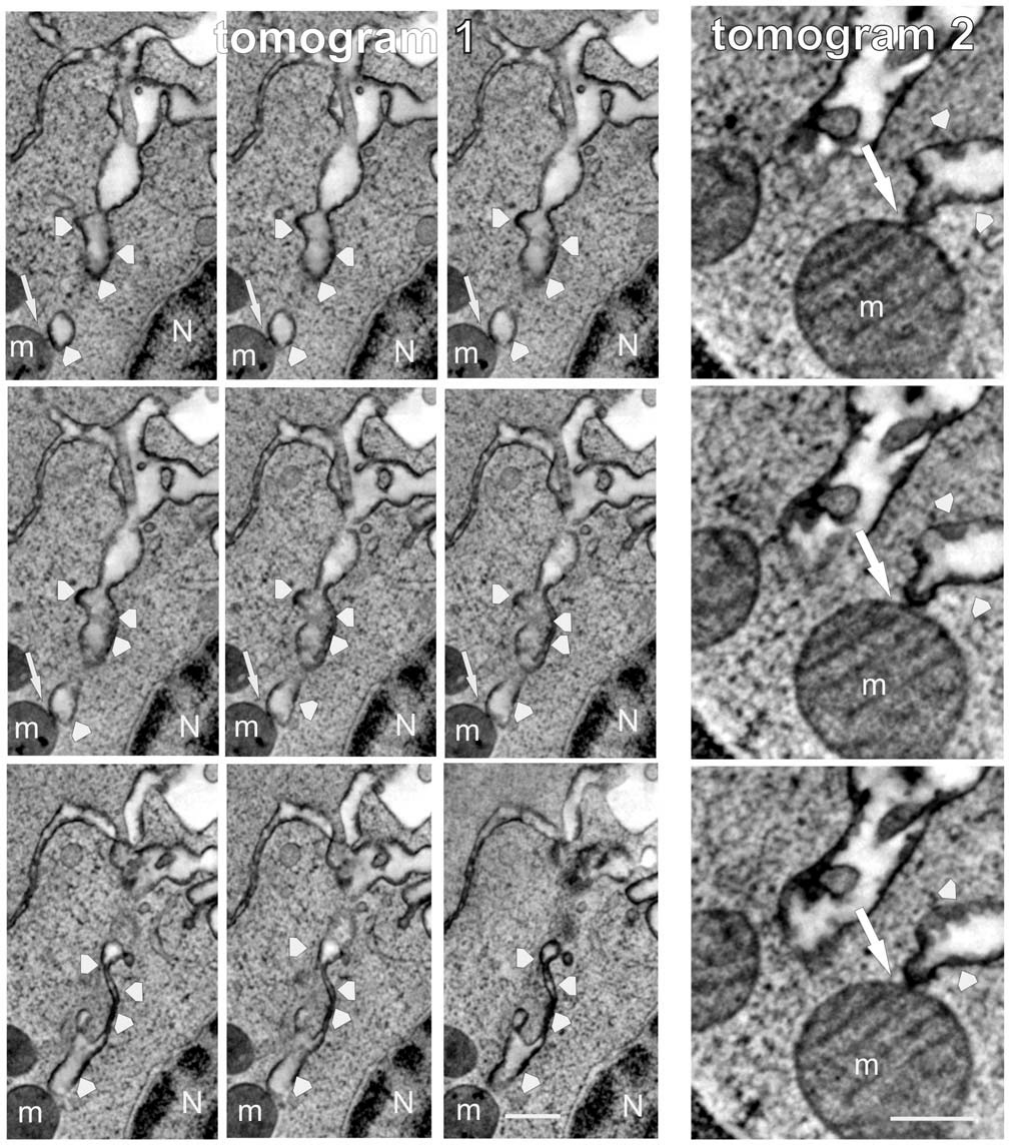
“Kiss-of-death” between the plasma membrane and mitochondria. Movie stills of electron micrographic tomograms of 2 different, unfixed, frozen Jurkat cells, whose outer leaflets of the plasma membrane were pre-labelled with horseradish peroxidase-cholera toxin B (arrowheads) before subjecting the cells to Ca^2+^ overload. Contact sites between surface-labelled membrane invaginations which extend deeply into the cytoplasm and mitochondrial outer membranes are visible (arrows). Mitochondrium (m), Nucleus (N). Bars = 0.5 µm.

To facilitate the structural identification of the sites of membrane invagination, the plasmalemmal surface was pre-labelled with the horseradish-peroxidase-tagged cholera toxin subunit B. Some of the horseradish-peroxidase-tagged, tube-like structures emanated from the plasma membrane, extended deep into the cytoplasm (Fig. 4e, boxed area enlarged in 4f, arrowhead) and ended in the vicinity of a mitochondrium (Fig. 4f, arrow). Electron microscopic tomography of 250 nm-thick, uncontrasted sections permitted a 3D-reconstruction of the invaginated plasmalemmal components (Fig. 5 arrowheads) which were observed to directly contact the mitochondrial outer membranes (Fig. 5 arrows, Movies S2 and S3). Plasmalemmal invaginations were found in 37.5% (±12% STD) of Jurkat cells in which Ca^2+^-overload had been elicited (n = 1800 of 6 independent experiments). Close proximity of plasmalemma and mitochondria (a distance of ≤0.2 µm between the plasmalemma and the outer mitochondrial membrane) was observed in 4.1% (±2.6% STD) of Jurkat cells. The distance of 200 nm was selected on the basis of micrographic tomography experiments that demonstrated that this distance was highly likely to lead to a direct contact and a physical apposition of the different membranes in the sections above or below the sectioned plane.

## Discussion

Apoptosis can be triggered by a receptor-mediated, extrinsic or by an intrinsic pathway. Both pathways are thought to be interlinked and converge on the mitochondria, whose outer membranes become increasingly permeable [31]. Consequently, proteins leaking out from the intermembranous space activate cytosolic caspases and DNAses, which ultimately lead to apoptotic cell death [32]. This increase in the permeability of the mitochondrial outer membrane has been ascribed to proteins and lipids alike. Firstly, the release of pro-apototic proteins from mitochondria can be mediated by Bax [1] which translocates to the mitochondria from the cytosol [3], [4]. Secondly, the increase in permeability occurs due to a local action of ceramide [6], [13], [33]. However, the mechanism by which ceramide appears in the mitochondrial membrane is not unequivocally established. Intriguingly, ceramide that is generated by the activity of acid sphingomyelinase within the plasma membrane is considered to be crucial for the regulation of apoptosis that is triggered by the activation of death receptors, cytotoxic drugs and environmental stress stimuli [14], [34], [35]. The central role of the plasmalemmal pool of ceramide (ceramide platforms) in the induction of apoptosis has been demonstrated in acid sphingomyelinase- (ASM) deficient cells and in ASM-deficient mice [36]–[38]. New data identify ceramide platforms as nucleation sites and import vehicles for cationic cell-penetrating peptides [39].

We have previously shown that plasmalemmal ceramide production, its self-association to membrane platforms and platform invagination/internalization is critically dependent on the elevation of intracellular Ca^2+^. No ceramide platform formation/invagination was observed in the presence of EGTA [18].

Here, we ascertained whether a stress-mediated plasmalemmal pool of ceramide might become available to the mitochondria of apoptotic cells. Using immunofluorescent microscopy we have confirmed an accumulation of ceramide within the mitochondria of fixed apoptotic cells. Using annexin A1 as a marker for ceramide platforms [18], we investigated structural changes in the plasma membrane of living cells undergoing Ca^2+^-overload-induced apoptosis. Extensive internalisation of the plasmalemmal annexin A1/ceramide platforms was observed and the invaginated plasma membrane established contacts with the mitochondrial outer membrane.

The most conclusive evidence for the establishment of direct contacts between the ceramide-rich plasmalemmal invaginations and mitochondria came from observations in the electron microscope. Fine structural analyses of dynamic membrane compartments are technically challenging. The preservation of cells by high-pressure freezing and freeze substitution permits a stabilisation of transient cell structures [24], [25] which cannot be achieved by chemical fixation. Using this technique, it has become possible to characterize more precisely not only major cytoskeletal structures, but also membranous systems and their dynamic relationships [40]. Labelling of the plasmalemmal surface permits an identification and visual tracing of internalized membranes. Our initial analysis of individual sections, revealed the apoptotic cells to display numerous, closed plasmalemma-derived vesicles. The generation of plasmalemmal infoldings and vesiculation is prevented by an inhibitor of acid sphingomyelinase, desipramine. But the full extent of the tubular invaginations was disclosed only by 3D electron microscopic tomography. Our data indicate that the vesicles comprise a communicating network of plasmalemmal invaginations which are rapidly formed in response to Ca^2+^-overload. And most importantly, these structures establish direct physical contacts with the mitochondrial outer membranes.

Massive, calcium-activated endocytosis, which did not involve any of the classical endocytic proteins (clathrin, dynamin, the actin cytoskeleton), has recently been described [17]. This type of “excessive” endocytosis, which might affect up to 25% of a cell's surface [17] reflects the formation of ceramide domains that develop high inward curvature and undergo spontaneous budding [41]. This phenomenon is probably analogous to the internalization of ceramide platforms visualized in our previous work [18]. Our present data highlights potential functional consequences which might arise from such a profound reorganisation of the plasmalemma elicited by [Ca^2+^]_i_ overload.

Proximity to plasmalemmal invagination, as determined by electron tomography was observed for 4.1% of mitochondria. And though the spatial resolution of this technique can hardly be surpassed, we opted to follow this dynamic process with a higher degree of temporal precision available in the confocal microscope. Plasmalemmal to mitochondrial proximity, as determined by colocalization of the respective markers in the confocal microscopy, occurred for ∼30% of mitochondria, regardless of the cell type or the method selected to induce Ca^2+^ overload.

Permeabilization of the outer mitochondrial membrane leads to a kinetically invariant, coordinate release of cytochrome c [42]. Yet, though this release was reported to be complete once it commenced, it did not occur simultaneously in all mitochondria, but appeared to affect different groups at different times. Indeed, the pattern depicted by Goldstein et al. [42] indicates that mitochondria, which are situated in the immediate vicinity of the plasma membrane are affected earlier than more centrally localized ones.

The close apposition of heterologous membranes has been observed to play a role in the transport of small molecules [43], and molecular markers for the contact sites are currently being identified [44]–[46]. Specialized contact sites between the endoplasmic reticulum (ER) or Golgi apparatus and mitochondria are important in the regulation of Ca^2+^-homeostasis [47]–[50]. The intracellular transport of lipids, which does not occur via vesicular routes, depends likewise on highly-selective transfer mechanisms. Accordingly, phosphatidylserine is transferred from the ER to the mitochondria via specific, mitochondrion-associated ER-membrane sites [51]. In the case of ceramide, both vesicular and non-vesicular shuttles between the ER and the Golgi apparatus have been described [52], [53]. The non-vesicular transport is mediated by the ceramide transfer protein (CERT) and depends upon the integrity of Golgi contact sites [54]. The direct contacts that we observed between the plasma membrane and the mitochondria described here highlights the role of interorganellar contacts in the transport of signalling molecules.

A murine mitochondria-associated neutral sphingomyelinase has been recently identified [10]. Interestingly, this enzyme is activated by phosphatidylserine [10], which is highly abundant on the inner leaflet of the plasma membrane. It is thus conceivable that – in addition to a direct transfer of ceramide – the phosphatidylserine-dependent activation of a mitochondrially-associated sphingomyelinase might trigger a local production of ceramide thereby permitting an efficient permeabilization of the outer mitochondrial membrane.

In conclusion, we have observed an accumulation of ceramide within the mitochondria of apoptotic cells and shown that ceramide platforms originating within the plasma membrane are being internalised and come into contact with the mitochondrial outer membrane. It is conceivable that ceramide is being exchanged at these contact points to trigger the release of pro-apoptotic molecules.

## Supporting Information

Movie S1 **AnnexinA1/ceramide platform dynamics in Jurkat T-cells after Ca^2+^-overload.** Time-lapse sequence of confocal microscopical images for a Jurkat T-cell that had been transiently transfected with annexin A1-YFP (yellow) and annexin A6 (blue), prior to stimulation with with SLO. Both annexins initially translocate to the plasma membrane. Thereafter, annexin A1 segregates from annexin A6 and coalesces into membrane platforms, which are internalised. The development of finger-like plasmalemmal invaginations of annexin A1 (arrows) is illustrated in Fig. 3.
(MOV)

Movie S2 **“Kiss of Death 1.”** Electron micrographic tomograms of unfixed, high-pressure frozen, freeze-substituted Jurkat T-cells, whose outer leaflets of the plasma membrane had been pre-labelled with the horseradish peroxidase-tagged cholera toxin B before stimulation with SLO. Sites of physical contact between surface-labelled membranous invaginations and the mitochondrial outer membranes are visible.
(MOV)

Movie S3 **“Kiss of Death 2.”** Electron micrographic tomograms of unfixed, high-pressure frozen, freeze-substituted Jurkat T-cells, whose outer leaflets of the plasma membrane had been pre-labelled with the horseradish peroxidase-tagged cholera toxin B before stimulation with SLO. Sites of physical contact between surface-labelled membranous invaginations and the mitochondrial outer membranes are visible.
(MOV)
